# Endoscopic and Pathologic Changes of the Upper Gastrointestinal Tract in Crohn's Disease

**DOI:** 10.1155/2014/610767

**Published:** 2014-02-03

**Authors:** Atsushi Sakuraba, Yasushi Iwao, Katsuyoshi Matsuoka, Makoto Naganuma, Haruhiko Ogata, Takanori Kanai, Toshifumi Hibi

**Affiliations:** ^1^Division of Gastroenterology and Hepatology, Department of Internal Medicine, Keio University School of Medicine, 35 Shinanomachi, Shinjyuku-ku, Tokyo 160-8582, Japan; ^2^Section of Gastroenterology, Hepatology and Nutrition, Department of Medicine, The University of Chicago, 5841 S. Maryland Avenue, MC 4076, Chicago, IL 60615, USA

## Abstract

*Background*. Crohn's disease (CD) may involve any part of the gastrointestinal tract. We assessed the prevalence and features of upper gastrointestinal (UGI) lesions in CD. *Methods*. This was a retrospective study that included 138 CD patients that underwent esophagogastroduodenoscopy (EGD). The rate of Crohn's specific endoscopic lesions in the esophagus, stomach, and duodenum was assessed, and immunohistochemical analysis was performed. Changes in the UGI lesions were assessed in those who had two or more EGD. *Results*. Of 138 patients, 51.3% had Crohn's specific UGI lesions. The rates of Crohn's specific lesion in the esophagus, upper-to-middle stomach, lower stomach, duodenal bulb, and 2nd portion of the duodenum were 6.5%, 47.8%, 24.6%, 31.9%, and 18.1%, respectively. Granulomas were detected in 6.1%, 25.0%, and 11.4% in the upper-to-middle stomach, lower stomach, and duodenal bulb, respectively, but none in the esophagus and 2nd portion of the duodenum. Thirty-seven were analyzed for *Helicobacter pylori* and 4 were positive (10.8%). Improvements of UGI lesions were seen in 14 out of 49 (28.5%) and were unchanged in 59.2% and worsened in 12.2%. *Conclusions*. The prevalence of Crohn's specific UGI lesions was common in our case series, and immunohistochemical studies suggested that the majority was unrelated to *Helicobacter pylori* infection. Worsening of UGI lesions over the course was rare.

## 1. Introduction

Crohn's disease (CD) is a chronic inflammatory bowel disease (IBD) characterized by periods of remission interrupted by the episodes of clinical relapse due to recurrent intestinal inflammation. The immunopathogenesis of CD reflects dysregulated interaction among environmental factors, intestinal flora, and genetic susceptibility factors within the immune system, which triggers inflammatory activities in the colonic mucosa [1]. CD typically affects the terminal ileum but may involve any part of the intestine. The upper gastrointestine, that is, esophagus, stomach, and duodenum, may also be involved [2], but the reported prevalence of upper gastrointestinal (UGI) lesions in CD has greatly varied due to the different definition and criteria of UGI lesions adopted in the studies [3, 4].

UGI lesions of CD include aphthae, erosions, ulcerations, bamboo joint like lesion, strictures, and notch like appearance [4, 5]; however, their prevalence among different regions in the UGI tract is unclear. While some case series report on the efficacy of treatment for UGI lesions of CD, their long term course remains unclear [6].

In the present study we first defined endoscopic UGI lesions of CD and assessed their prevalence in a cohort of CD patients. The rates of *Helicobacter pylori *infection and granuloma detection were analyzed, and for those who had multiple esophagogastroduodenoscopy (EGD), the changes in the UGI lesions were investigated.

## 2. Methods

### 2.1. Participants and EGD

This was a retrospective cohort study, which aimed to describe the prevalence and features of UGI lesions in patients with CD who underwent EGD from January 2001 to January 2006 at Keio University Hospital. Case files of 138 patients who underwent EGD were reviewed for demographics, CD characteristics, comorbidities, and concomitant CD treatments (duration and dosages). EGD was performed as routine diagnostic procedure of CD, when patients had UGI symptoms such as epigastric pain, reflux, and nausea, or when considered necessary by the physician based upon patient background.

### 2.2. Outcomes

**Figure 1:**
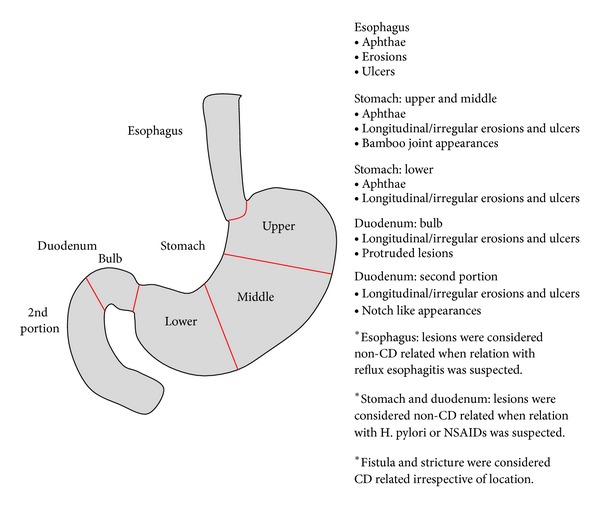
Definition of UGI lesions specific to CD.

The prevalence of endoscopic findings in the esophagus, stomach, and duodenum was assessed. Lesions specific to CD were defined as in Figure 1. In the esophagus, aphthae, erosions, ulcers, and strictures that appeared unrelated to reflux esophagitis were regarded as CD specific. The stomach was assessed in two lesions, mid-upper stomach (body-fundus) and lower stomach (antrum), and aphthae, longitudinal/irregular erosions and ulcers, fistulas, and bamboo joint like appearance were considered as CD specific [7]. In the duodenal bulb, longitudinal or irregular erosions and ulcers, and protruded lesions were considered as CD specific. In the 2nd portion of the duodenum, longitudinal/irregular erosions and ulcers, and notch like appearances were considered as CD specific. Erythema, erosions, and ulcers that improved with proton pump inhibitor treatment were excluded. Lesions were considered non-CD related when relation with  *Helicobacter pylori* or nonsteroidal anti-inflammatory drugs(NSAIDs) was suspected.

Biopsy samples were read by a single pathologist at the department of pathology. The prevalence of *Helicobacter pylori* was assessed by immunohistochemistry using rabbit anti-*Helicobacter pylori* antibody (DAKO, Denmark) with appropriate positive and negative controls. Biopsies of the greater curvature of the upper to mid and lower stomach were processed separately.

For those who had two or more EGD, the changes in the UGI lesions were assessed.

### 2.3. Statistical Methods

For statistical analysis, data were processed by SAS software (SAS Institute, Cary, NC, USA). Standard descriptive statistics, such as mean, median, and standard deviations, were computed for continuous variables. All *P* values are two-sided and *P* < 0.05 was considered statistically significant.

## 3. Results

### 3.1. Participants

A total of 138 CD patients that underwent EGD during the study period were identified. The diagnosis of CD was made by a combination of endoscopic, histologic, and radiologic studies in all patients using conventional criteria [8]. The characteristics of patients are shown in Table 1. One hundred and three were males and thirty five were females, and average age was 35.4 (range 11–74). Indication of EGD was UGI symptoms such as nausea and epigastric pain in 35, and in the rest it was done by part of work-up. Forty-nine patients had two or more EGD at least 6 months apart, and changes of endoscopic findings were assessed.

### 3.2. Prevalence of Upper GI Lesions

**Figure 2:**
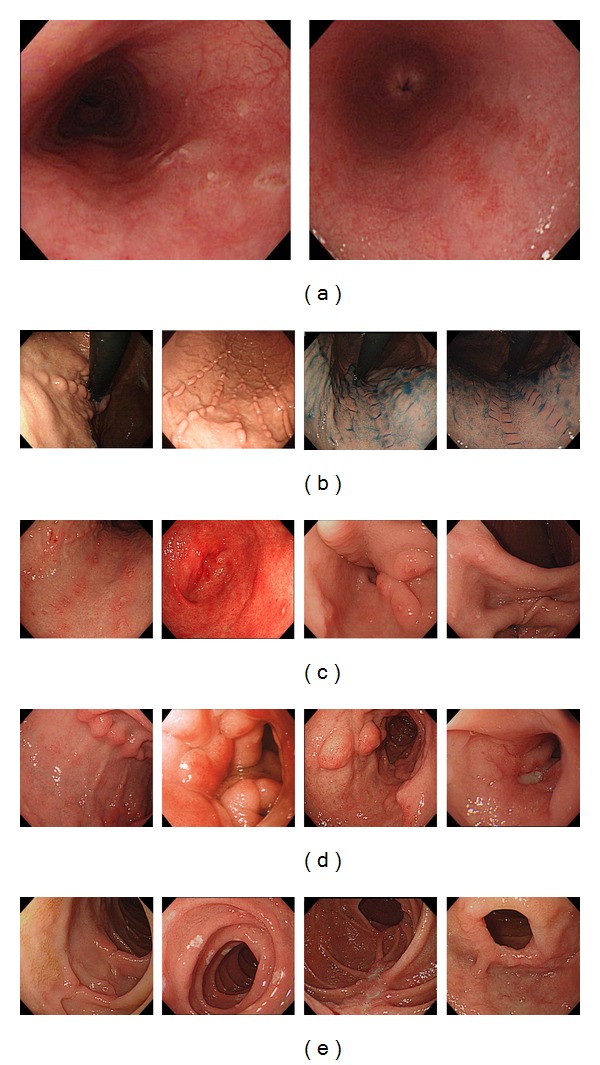
Endoscopic findings of UGI lesions specific to CD. (a) Esophagus, (b) upper to mid stomach, (c) lower stomach, (d) duodenal bulb, and (e) 2nd portion of duodenum.

As shown in Table 2, the rates of detecting Crohn's specific lesion by endoscopy in the esophagus, upper to middle stomach, lower stomach, duodenal bulb, and 2nd portion of the duodenum were 6.5%, 47.8%, 24.6%, 31.9%, and 18.1%, respectively. Lesions that were considered as non-CD related included reflux esophagitis, hiatal hernia, candida esophagitis, erythema, and erosions that could be attributed to the use of nonsteroidal anti-inflammatory drugs based on appearance and histology. We defined the aphthae, erosions, or ulcers in the esophagus as Crohn's disease related when they were located far from the gastroesophageal junction or were present when there was no evidence of reflux esophagitis or when histology showed inflammation consistent with Crohn's disease. Gastric and duodenal erosions/ulcers that improved with proton pump inhibitor treatment were excluded as well. Pictures of CD related lesions are shown in Figure 2. There was no difference in regard to age, disease duration, location of disease, and the presence of perianal lesions among those with or without UGI lesions (data not shown).

### 3.3. Detection of Granulomas

Noncaseating granulomas were detected in 15 out of 138 (10.9%) of patients (Table 2). They were detected in 6.1%, 25.0%, and 11.4% in the upper to middle stomach, lower stomach, and duodenal bulb, respectively. Interestingly, no granulomas were detected in the esophagus and 2nd portion of the duodenum, in our patient population. This may be due to low detection rate of CD specific lesions in the esophagus and 2nd portion of the duodenum as compared to the stomach and bulb. However, the numbers and locations of biopsies may well affect the detection rate of granulomas as well.

### 3.4. *Helicobacter pylori* Infection

Thirty-seven patients were analyzed for *Helicobacter pylori* infection by immunohistochemistry. Four out of thirty-seven (10.8%) were positive for *Helicobacter pylori*, which was comparable to previous reports.

### 3.5. Changes in the UGI Lesions

**Figure 3:**
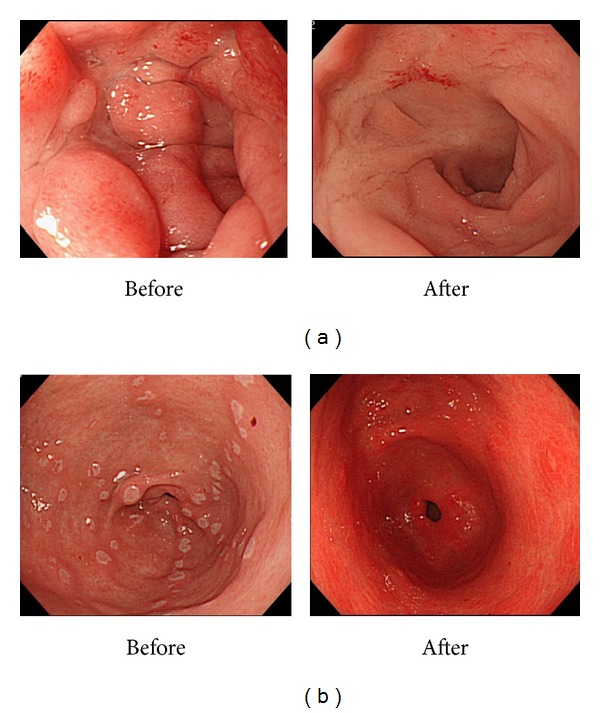
Changes in the endoscopic findings during the clinical course. (a) Improvement of duodenal bulb lesion with mesalamine treatment. (b) Improvement of antral erosion/ulceration with mercaptopurine treatment.

The clinical changes in the UGI lesions were assessed in those who had two or more EGD. Forty-nine patients met this criterion. The average time to the second EGD was 16.4 months (range of 6–32 months). Improvements of UGI lesions were seen in 14 out of 49 (28.5%) but were unchanged in 29 (59.2%) and worsened in 6 (12.2%). Of the 14 who had improvement of the UGI lesions, six were attributed to the use of mesalamine or immunomodulator (Figure 3), and in the rest the lesions saw improvement without the addition of specific CD treatment or the patient received multiple medications. Of the 6 that experienced worsening of the lesions, two required gastrojejunal bypass surgery due to a severe stricture of the duodenum. In many occasions the patient received multiple medications and response to specific treatment could not be analyzed. Overall, it can be interpreted that UGI lesions remain rather stable during the time course, and complications are quite rare as compared to ileal or colonic disease.

## 4. Discussion

In the present study we have shown that more than half of the CD patients that underwent EGD had macroscopic UGI lesions that appeared to be specific to CD. Infection with *Helicobacter pylori* was rare, whereas the detection of granuloma was relatively common. Worsening of the UGI lesions was rare and in the majority of patients they remained unchanged.

CD mainly affects the ileum and colon, but more recently UGI involvement has gained attention [1, 9, 10]. The reported rate of UGI lesions in CD varies from 1% to about 80%. The difference in the rate of UGI involvement is more likely due to the different criteria that were adopted among the studies. Some studies used histology whereas some studies used macroscopic criteria, like we did. The difference in the age of the patient population may also affect the outcome [11]. However, we presume that the adopted criterion is the largest factor affecting the rate of UGI lesions, rather than age, ethnic background, or other factors.

The proportion of patients positive for *Helicobacter pylori* infection was low in our population, which is similar to the previous studies [5]. This has been attributed to the frequent antibiotic use in patients with CD. Another possibility is that patients with Crohn's disease have less incidence of various infections referred to as the hygiene hypothesis. The frequency of identification of granulomas in our study was similar to the previous studies [5, 12]. We assume that a similar pathogenic process as in the ileum and colon is taking place in the UGI lesions of CD patients. In the present study, we assessed the rate of granuloma detection, instead of focally enhanced gastritis or the severity of gastritis [4]. The clinical significance of focally enhanced gastritis in CD varies between researchers [13], and, thus, we analyzed the frequency of granulomas as we thought that they serve as a better histologic marker in CD. The detection of granulomas may vary largely depending on the location and numbers of biopsies and specimen analyzed; however, our results were similar to the ones previously reported.

We also analyzed the clinical changes in the UGI lesions for those patients that underwent two or more EGD. We showed that only 12.2% saw worsening of the UGI lesions and among them only 2 required surgical intervention. It was suggested that UGI lesions remain relatively stable during the disease course of CD; however, as not all patients had a repeat EGD, the precise rates cannot be drawn from our study.

Some studies have suggested that the findings on EGD can be used to discriminate between CD and ulcerative colitis [14]. We did not have a control group, such as healthy controls or patients with ulcerative colitis, in our study; however, since granulomas can often be identified, we suggest that EGD and UGI findings are informative tools in the diagnosis of IBD. However, the utility and necessity of EGD in the diagnosis of IBD, especially CD, need to be validated in larger prospective studies. EGD has been more utilized in pediatric IBD population, but only a minor fraction of our patients had a pediatric onset and we could not compare the results between the pediatric and adult onset patients.

The shortcomings of our study are that we only analyzed data of those patients who underwent EGD in a retrospective fashion, *Helicobacter pylori *was analyzed in only one third of patients, and histological assessment was limited to granuloma detection. Our patient population may not represent the general CD population as the indication for EGD was not consistent (i.e., UGI symptoms or part of work-up). Immunohistochemistry of *Helicobacter pylori *was done only when there were lesions suspicious for *Helicobacter pylori* involvement, which may also not represent the true incidence in the general CD population. We did not include other histological assessments, such as focally enhanced gastritis, as there remains controversy whether they are specific to CD.

In conclusion, we have shown that UGI lesions are common among CD patients based upon our endoscopic criteria but often remain stable during the disease course. The detection of granuloma was relatively common in the UGI, suggesting that a similar disease process as in the ileum/colon is taking place in the UGI.

## Figures and Tables

**Table 1:** Patient characteristics.

Characteristics (*n* = 138)	
Sex (male : female)	103 : 35
Age (yr; range)	35.4 (11–74)
Duration of CD (yr)	12.2 ± 7.4
Disease location (ileum : ileocolonic : colonic)	31 : 92 : 15
Anal lesions (*n*)	45
Use of 5-ASA (*n*)	121
Use of steroids (*n*)	28
Use of immunomodulators (azathioprine/mercaptopurine) (*n*)	35
Use of infliximab (*n*)	10
Use of proton pump inhibitors (*n*)	10
Smoker (*n*)	19

**Table 2:** Frequency of identification of characteristic findings and granulomas.

Location	Any findings	CD specific findings	Granuloma detection
Esophagus	19 (13.8)	9 (6.5)	0/9 (0.0)
Stomach: upper to mid	71 (51.4)	66 (47.8)	4/66 (6.1)
Stomach: lower	83 (60.1)	24 (24.6)	6/24 (25.0)
Duodenum: bulb	58 (42.0)	44 (31.9)	5/44 (11.4)
Duodenum: 2nd portion	26 (18.8)	25 (18.1)	0/25 (0.0)

Total (*n* = 138)	104 (75.4)	70 (51.3)	15/138 (10.9)

( ): %.

## Abbreviations

CD: Crohn's disease
IBD: Inflammatory bowel disease
UGI: Upper gastrointestinal.
